# Interconnection between flowering time control and activation of systemic acquired resistance

**DOI:** 10.3389/fpls.2015.00174

**Published:** 2015-03-19

**Authors:** Zeeshan Z. Banday, Ashis K. Nandi

**Affiliations:** School of Life Sciences, Jawaharlal Nehru UniversityNew Delhi, India

**Keywords:** SAR, flowering, SA, FLD, chromatin remodeling, epigenetic

## Abstract

The ability to avoid or neutralize pathogens is inherent to all higher organisms including plants. Plants recognize pathogens through receptors, and mount resistance against the intruders, with the help of well-elaborated defense arsenal. In response to some localinfections, plants develop systemic acquired resistance (SAR), which provides heightened resistance during subsequent infections. Infected tissues generate mobile signaling molecules that travel to the systemic tissues, where they epigenetically modify expression o a set of genes to initiate the manifestation of SAR in distant tissues. Immune responses are largely regulated at transcriptional level. Flowering is a developmental transition that occurs as a result of the coordinated action of large numbers of transcription factors that respond to intrinsic signals and environmental conditions. The plant hormone salicylic acid (SA) which is required for SAR activation positively regulates flowering. Certain components of chromatin remodeling complexes that are recruited for suppression of precocious flowering are also involved in suppression of SAR in healthy plants. FLOWERING LOCUS D, a putative histone demethylase positively regulates SAR manifestation and flowering transition in *Arabidopsis*. Similarly, incorporation of histone variant H2A.Z in nucleosomes mediated by PHOTOPERIOD-INDEPENDENT EARLY FLOWERING 1, an ortholog of yeast chromatin remodeling complex SWR1, concomitantly influences SAR and flowering time. SUMO conjugation and deconjugation mechanisms also similarly affect SAR and flowering in an SA-dependent manner. The evidences suggest a common underlying regulatory mechanism for activation of SAR and flowering in plants.

## Introduction

Immobility precludes plants from evading pathogens. However, the presence of strong immune system most often keeps them healthy. Higher animals like vertebrates, are capable of retaining an infection memory with the help of dedicated immune system and circulatory cells. The generated infection memory facilitates stronger immune response during subsequent interactions with the same pathogen (adaptive immunity). Despite not having dedicated immune cells, plants are equally capable of using infection-induced molecular memories to resist subsequent infections. This heightened resistance based on past experience is called systemic acquired resistance (SAR; Ross, 1961). Unlike adaptive immunity-based learning in animals, SAR-mediated protection in plants is not limited to the same pathogen, but is effective against a wide range of microbial pathogens (Sticher et al., 1997; Durrant and Dong, 2004).

While pre-existing cell wall and structural components such as cuticular wax provide resistance against pathogens, most defense responses are induced upon pathogen infection. Resistance against pathogens in plants relies both on fortification of structural barriers and production of antimicrobial chemicals and proteins. Microbe/pathogen associated molecular patterns (MAMPs/PAMPs) are recognized by the plasma membrane (PM) resident pattern recognition receptors (PRRs; Nimchuk et al., 2003). Recognition of MAMP/PAMP by PRRs activates signaling cascades involving kinases, proteases, protein modifiers and transcription regulators, which eventually results in cell wall strengthening, production of antimicrobial proteins and phytoalexins (Schwessinger and Ronald, 2012). The defense hormones such as salicylic acid (SA), ethylene (ET), and jasmonic acid (JA) function as second messengers of the signaling events (Verhage et al., 2010). PRR activation induces biosynthesis of these hormones, which in turn leads to transcriptional reprogramming in favor of defense. Pathogen infection induces transcription of large number of genes, a subset of these are *pathogenesis related* (*PR*) genes (van Loon et al., 2006). Several PR proteins are secreted out of the host cell and negatively affect the growth of pathogens due to their antimicrobial properties (van Loon et al., 2006). Immune responses as described above, triggered by activation of PRRs is known as pattern triggered immunity (PTI). Some pathogens, however, release effector molecules to suppress the plant PTI response (Jones and Dangl, 2006). Plants can overcome the effects of pathogen effectors using R gene-mediated resistance, in which R receptors interact directly or indirectly with pathogen effectors to initiate effector-triggered immunity (ETI). ETI is an exaggerated form of PTI (Jones and Dangl, 2006).

**FIGURE 1:**
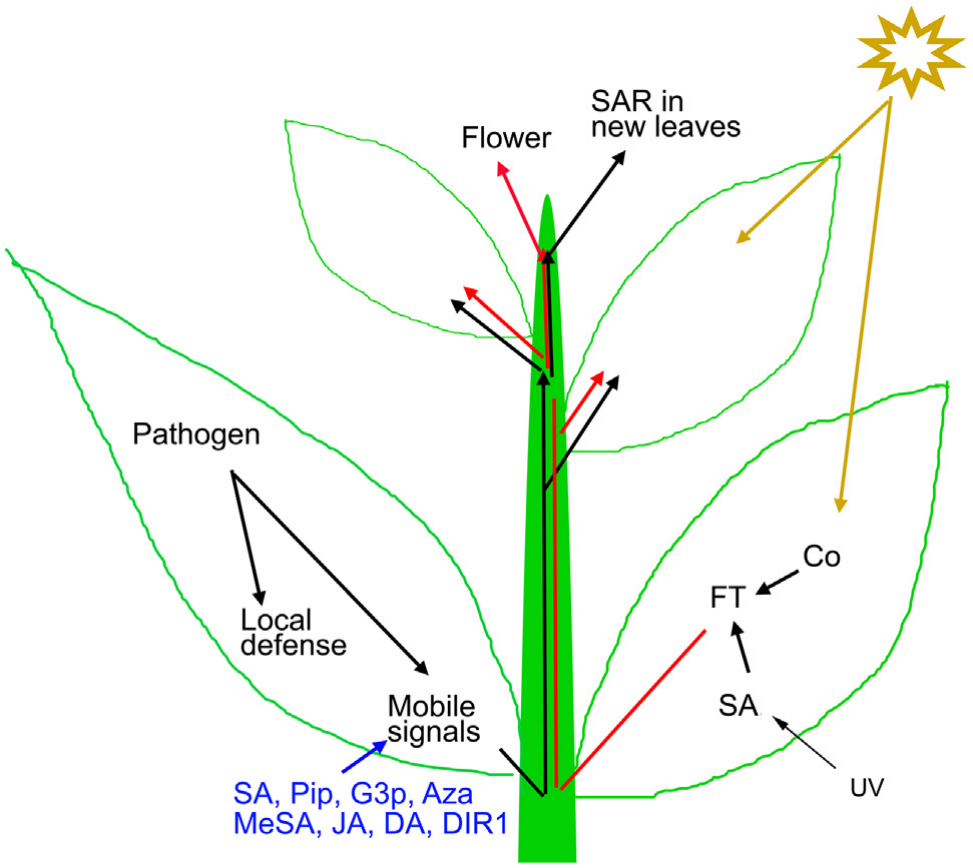
**Mechanism of SAR and flowering induction.** Local pathogen inoculation induces local defense and mobile SAR signals (such as SA, Pip, G3P, Aza, MeSA, JA, DA, and DIR1) that travel to distal parts through phloem for SAR activation. The generated SAR signals are capable of SAR induction in leaves that develop after the primary induction (Caruso and Kuc, 1977). Exogenous application of these mobile signals also induces resistance throughout the plant. Light quality and quantity influences floral regulators like FT that also travels through the phloem to modify the shoot apex to produce flowers instead of leaves.

When a plant succeeds in restricting the growth of a pathogen, it develops SAR; a state of preparedness that provides elevated resistance during subsequent infections (Durrant and Dong, 2004; Iriti and Faoro, 2007; Vlot et al., 2008). Besides pathogens, certain chemicals such as SA and its chemical analogs are capable of inducing SAR in plants (Lawton et al., 1996). During the SAR inducing infection, mobile signals are synthesized in the infected tissue and get distributed throughout the plant, via phloem (**Figure 1**; Guedes et al., 1980; Tuzun and Kuc, 1985). It has been demonstrated that upon localized pathogen inoculation, the pathogen free distal tissues show immune responses like the infected tissues, but to a moderate level. For example, distal tissues show fortification of cell wall, accumulation defense hormones and expression of PR-proteins (Ward et al., 1991; Ryals et al., 1996; Fu and Dong, 2013). But more importantly, an experienced plant activates priming, a SAR induced mechanism that results in robust induction of defense responses compared to a naive plant, during subsequent pathogen infections (Jung et al., 2009; Slaughter et al., 2012; Singh et al., 2013). Genetic and biochemical experiments, mostly on model plants, identified several compounds such as SA, methyl salicylate, JA, dihydroabetinal, azelaic acid, glycerol-3-phosphate, pipecolic acid, and lipid transfer protein DIR1 as potential mobile signals of SAR (Dempsey and Klessig, 2012; Navarova et al., 2012; Champigny et al., 2013). Petiole exudates enriched for phloem sap collected from pathogen-inoculated leaves, carrying these mobile signals are capable of inducing SAR in naive plants (Chaturvedi et al., 2008; Chanda et al., 2011).

Mechanism of the development of infection memory, subsequent to receiving the mobile signal in distal tissues, is not elucidated well. The mobile signals by themselves are not antimicrobial (Jung et al., 2009; Chanda et al., 2011; Chaturvedi et al., 2012; Navarova et al., 2012). The metabolic signals do not directly provide SAR, as they are elevated only transiently, while SAR lasts for weeks to months as observed in cucumber, watermelon, muskmelon, and other plants (Caruso and Kuc, 1977; Kuc and Richmond, 1977). Thus, for the induction of SAR, the systemic tissues must perceive and decode the SAR signals. Recent studies, mostly with the model plant *Arabidopsis* provide evidence that upon infection, epigenetic modifications takes place in systemic tissues, which contribute to infection memory formation. Promoters of the plant specific WRKY transcription factors have been reported to accumulate elevated levels of modified histones that are normally associated with epigenetic control of gene expression (Jaskiewicz et al., 2011; Luna et al., 2012; Singh et al., 2014b). Modified histones on WRKY genes involved in SAR could be part of infection memory. It’s not clear how this epigenetic mechanism relates to SAR memory.

**FIGURE 2:**
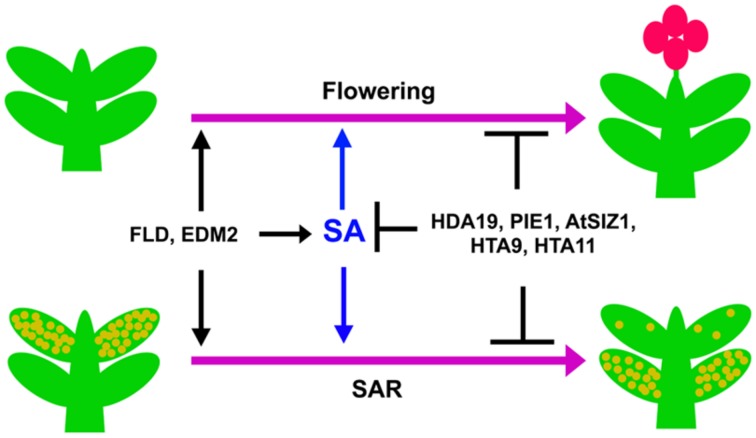
**Transition from vegetative stage to reproductive stage and development of SAR is controlled by SA.** Upon attaining the right developmental stage, plants show transition from the vegetative to the reproductive phase of growth (upper two plants). A plant that has previously experienced a pathogen develops fewer disease symptoms after subsequent infections due to SAR (lower right plant) compared to an inexperienced plant (lower left plant). SA positively influences both of these processes. The genes mentioned in the figure similarly affect SA accumulation, flowering and SAR.

Recent studies indicate a close interconnection between flowering time control and SAR activation mechanisms. The transition to flowering is an irreversible process for annual plants, when the shoot apical meristem becomes an inflorescence meristem that produces flowers instead of leaves. The timing of this transition is a major factor for the reproductive success of plants. Regulation of flowering time involves complex regulatory network consisting of multiple set of genes (Simpson and Dean, 2002). The flowering molecular switch ensures that plants flower at a time when internal resources are adequate and the ambient environmental conditions are optimum for pollination and seed development (Simpson et al., 1999). A large number of gene products affect both flowering and SAR (**Figure 2**). This review article discusses the possible mechanistic overlap in regulation of flowering time and SAR.

## Flowering Control by Salicylic Acid and other SAR Inducers

Functions of SA and its derivatives are intricately associated with SAR. SA and its chemical analogs are potential SAR inducers when exogenously applied to plants (Yalpani et al., 1991; Gaffney et al., 1993). When a plant is infected by a pathogen, high level of SA accumulates in the pathogen-infected tissue and to a lesser extent in pathogen free systemic tissues (Metraux et al., 1990; Nandi et al., 2004). SA promotes nuclear localization and activation of NON-EXPRESSOR OF PR-1 (NPR1), a *trans*-activator protein, which is required for SAR (Kinkema et al., 2000; Wu et al., 2012). NPR1 interacts with TGA transcription factors, and together induce expression of *PR* genes (Dong, 2004). Expression of PR*-1* gene is typically associated with the activation of SA signaling and thus serves as its marker. The mutants such as *suppressor of fatty acid desaturase 1* (*sfd1*), *reduced systemic immunity 1* (*rsi1*), *azelaic acid induced 1* (*azi1*) of *Arabidopsis* that are impaired in SAR induction are defective in systemic SA accumulation, and priming induced expression of *PR-1* (Chaturvedi et al., 2008; Jung et al., 2009; Singh et al., 2013). SA has been implicated as an integral component of SAR signaling (Ryals et al., 1996; Sticher et al., 1997; Conrath, 2011; Fu and Dong, 2013).

Interestingly, SA also influences flowering time to a great extent. Involvement of SA in common regulation of SAR/pathogen response and flowering is reflected in many reports (discussed in the following sections; ). *ENHANCED DOWNY MILDEW 2* (*EDM2*) gene of *Arabidopsis* is required for *RPP7*-mediated resistance against downy mildew pathogen *Hyaloperonospora parasitica* (Eulgem et al., 2007). The mutants of EDM2 fail to accumulate pathogen induced SA, and also cause flowering time delay (Tsuchiya and Eulgem, 2010).

### Effect of Light on SA, SAR, and Flowering

Light plays very important role in biosynthesis of SA and immune responses (Zeier et al., 2004; Kangasjarvi et al., 2012). Pathogen induced SA biosynthesis takes place in chloroplast, in light (UV-C) dependent manner (Fragniere et al., 2011). A large number of genes that are induced upon flg22 (bacterial flagellin derived PAMP peptide) treatment require light (Sano et al., 2014). Light composition, intensity, and duration affect defense responses (Chandra-Shekara et al., 2006; Griebel and Zeier, 2008; Ballare et al., 2012; de Wit et al., 2013). Red light stimulates disease resistance against many pathogens (Islam et al., 2008; Wang et al., 2010). In contrast, addition of far-red light (leading to reduced red:far-red ratio) negatively influences defense responses (de Wit et al., 2013). Plants perceive red and far-red lights by two inter-convertible forms of phytochrome photo-receptors, Pr and Pfr, which absorb red and far-red light, respectively. The Pr is the inactive form, which converts into the active form Pfr, upon absorbing red light, whereas, the Pfr form converts back into the Pr form by absorbing far-red light (Smith, 2000). The *phyAphyB* double mutant plants are susceptible against virulent pathogen *Pseudomonas syringae* pv. *maculicola* ES4326 (Psm; Griebel and Zeier, 2008). The results suggest that phytochrome signaling plays a very significant role in disease defense. Does phytochrome signaling have any specific role in SAR? The experimental evidence is insufficient at the present time to draw this conclusion. Griebel and Zeier, 2008, reported that the phytochrome signaling is more pertinent for SAR than local defense. However, this conclusion may be accepted with certain reservations. The *phyAphyB* mutant plants are highly susceptible to Psm, and support modestly higher growth of Psm carrying the avirulence gene *avrRpm1* (Psm-AvrRpm1) compared to wild-type plants. The *phyAphyB* plants, but not the mutants of other photoreceptors such as cryptochromes (*cryAcryB*) and phototropins (*phot1 phot2*) are defective activation of SAR. Surprisingly, authors used Psm as primary pathogen for SAR induction, against which *phyAphyB* plants were compromised for local defense, instead of Psm-AvRpm1 (Griebel and Zeier, 2008). Moreover, other studies, such as the effect of red light in promoting disease defense, and low red-far red ratio affecting general defense responses, also counter argues for phytochromes having specific roles in SAR.

The role of light in flowering is much well-established. Amongst the environmental factors that affect flowering, light plays the most important role. According to the photoperiod dependence for flowering, angiosperms are grouped into long-day (LD), short-day (SD), and day-neutral plants. *Arabidopsis* is a facultative LD plant that flowers early in LD, and show delayed flowering under SD condition. The striking similarity between SAR and photo-period induced flowering is the requirement of long distance signal movement through phloem (**Figure 2**; Zeevaart, 2006). Grafting and girdling experiments suggested that the flowering inducers are phloem transmissible (Knott, 1934; Chailakhyan, 1936). By the perception of the day-length effect, leaves generate a mobile signal for flowering. The signal moves to the growing apex via phloem; the apex modifies to produce flower instead of leaves (Knott, 1934; Chailakhyan, 1936). In recent years, it has been shown that the phloem mobile flowering promoting factor is a protein; *flowering locus T* (FT) in *Arabidopsis* (Corbesier et al., 2007). FT orthologs have been identified in many plants suggesting that the vascular conductance is a universal feature for flowering in plants (Tamaki et al., 2007; Varkonyi-Gasic et al., 2013; Li et al., 2014).

Interestingly, SA is also reported as an inducer of photoperiod-mediated flowering. Abiotic stress such as UV-C induces expression of *FT* as well as flowering in SA dependent manner in *Arabidopsis* (Martinez et al., 2004). The transgenic plants expressing NahG fail to induce *FT* expression and early flowering by UV-C treatment (Martinez et al., 2004). The phloem sap, or the honeydew produced by aphid infestation, on *Xanthium strumarium* is capable of inducing flowering in the long-day plant *Lemna gibba* (Cleland and Ajami, 1974). Purification of flowering inducing component from aphid-honeydew by TLC, followed by GLC and mass-spectrometric analysis identified SA as the active ingredient of flowering inducer in phloem sap (Cleland and Ajami, 1974). Exogenous application of SA in the growing medium or in leaves, in several plants promote flowering (Cleland and Ajami, 1974; Khurana and Cleland, 1992; Wada et al., 2014). Thus, SA may be considered as a common inducer for both flowering and SAR (). A similar dual role is also reported for pipecolic acid (Pip), another mobile signal for SAR induction (Navarova et al., 2012). Flowering inducing activity guided fractionation identified Pip and nicotinamide as flowering inducing substances in *L. gibba* leaf extracts (Fujioka et al., 1987).

## FLD Regulates the Transition to Flowering and SAR

At a defined time in their life-cycle, annual plants undergo a developmental transition from the vegetative to the reproductive stage. This transition is controlled by environmental as well as endogenous developmental cues. The environmental factors include day length (photoperiod), quality and quantity of light (composition and photon density), prolonged cold exposure (vernalization), and nutrient and water availability, whereas, plant age and vegetative growth provide developmental cues for transition (see Amasino, 1996; Aukerman and Amasino, 1996). In *Arabidopsis*, mutational analysis has identified numerous genes that affect flowering time. The CONSTANS protein accumulates in long-days and positively regulates expression of *FT*, and SUPPRESOR OF CO 1 (SOC1), and thereby promotes flowering (Suarez-Lopez et al., 2001). In contrast, *FLOWERING LOCUS C* (*FLC*) negatively regulates *FT* and SOC1, and helps plants to avoid premature flowering (Michaels and Amasino, 1999; Helliwell et al., 2006). FLC codes for a MADS box protein that binds to promoters of FT and SOC1 repressing transcription of these genes (Helliwell et al., 2006). A large number of genes, whose expression is modulated by developmental cues and environmental factors, affect expression of *FLC* and control flowering time (Henderson and Dean, 2004). The FLC locus is epigenetically regulated through histone modifications. FLOWERING LOCUS D (FLD) negatively regulates expression of *FLC* and thereby promotes flowering (He et al., 2003; He and Amasino, 2005; Liu et al., 2007). Thus, flowering is delayed in *fld* loss-of-function mutants (He et al., 2003; Singh et al., 2013).

**FIGURE 3:**
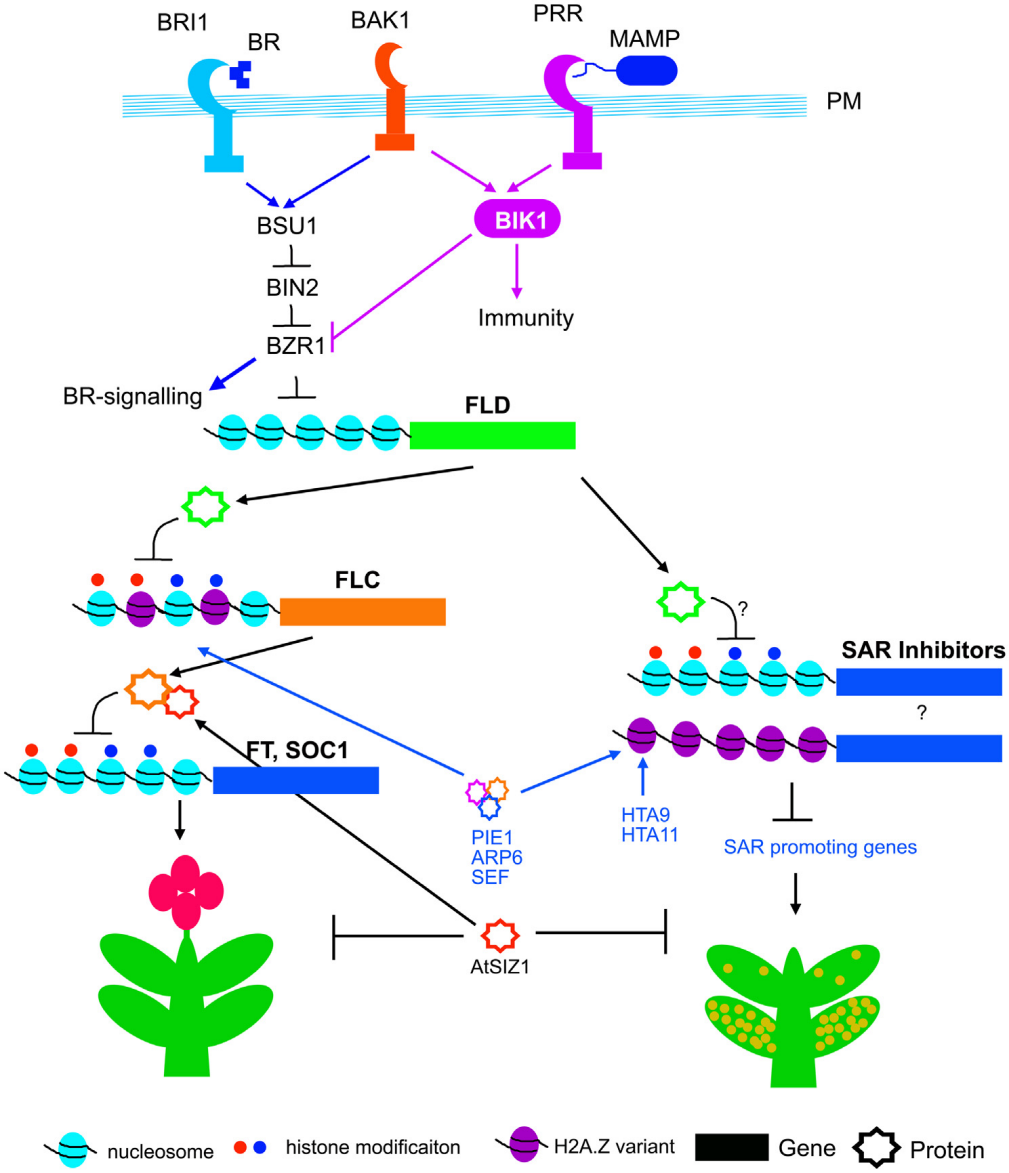
**Genetic and epigenetic control of flowering and SAR.** Plasma membrane (PM) resident BAK1 associates with both BRI1 and PRRs (pattern recognition receptors) which are required for BR and PTI signaling respectively. BSU1 phosphatase is activated by BRI1 and BAK1. BSU1 dephosphorylates and inactivates BIN2, and thereby activates BZR1 and BR signaling. BZR1 negatively regulates FLD expression. Activation of PTI activates BIK1, which suppress BZR1 and thereby may promote expression of FLD. FLD transcriptionally suppress FLC, the floral repressor. FLC protein is stabilized through interaction with AtSIZ1. AtSIZ1 functions as negative regulator for both flowering and SAR. The PIE1, ARP6 and SEF complex, and HTA9, HTA11 promote histone variant incorporation and biosynthesis, and thus promote transcription of FLC and unknown SAR suppressors.

A genetic screen selecting for SAR-impaired mutants from EMS treated *Arabidopsis* plants, identified *reduced in systemic immunity 1* (*rsi1*), which is a loss-of-function allele of *FLD* (Singh et al., 2013). The *rsi1* mutant is defective in systemic accumulation of SA and priming of *PR-1*, *WRKY6,* and *WRKY29* genes (Singh et al., 2013, 2014b). Petiole exudates from inoculated *rsi1* leaves activate SAR on WT plants, whereas, SAR inducible petiole exudates from WT plants fail to induce SAR in *rsi1.* Moreover, SAR is not induced in *rsi1* plants by exogenous application of SAR inducers such as dihydroabetinal and azelaic acid. Thus, the *rsi1* and the allelic *fld* mutants are capable of generating SAR mobile signals after primary infection, but fail to decode the signal in the distal tissues. These data suggest that FLOWERING LOCUS D function is required for generating infection memory, subsequent to receiving the SAR signal. FLD expression is induced both in the primary SAR-induced and systemic tissues (Singh et al., 2013). As a consequence, FLC expression maybe suppressed in by SAR induction. Indeed, transcript analysis following SA treatment showed suppression of FLC expression (Martinez et al., 2004). Although, *FLC* expression is suppressed by SA, its function is probably not associated with SAR (Singh et al., 2013). The *flc* mutant has no defect in SAR activation, and the *flc* mutation does not rescue the SAR defect in the *rsi1*/*fld* mutant. Thus FLD may function as branch point between flowering time control and SAR activation in *Arabidopsis* (**Figure 3**).

The mechanism of FLD expression in response to SAR induction is not known. Brassinosteroid (BR) signaling has recently been associated with FLD expression (Zhang et al., 2013b). BR is perceived by the receptor kinase, BR INSENSITIVE1 (BRI1) along with BRI1 ASSOCIATED KINASE1 (BAK1; Yang et al., 2011). Binding of BR activates both BAK1 and BRI1 through auto- and *trans*-phosphorylation, which in turn release the receptor-like cytoplasmic kinases BRASSINOSTEROID SIGNALING KINASES (BSKs) and CONSTITUTIVE DIFFERENTIAL GROWTH1 (CDG1; Tang et al., 2008; Kim et al., 2011). BSKs and CDG1 phosphorylate and activate BRI1 SUPPRESSOR1 (BSU1), a phosphatase that dephosphorylates BIN2 (Clouse, 2011; Kim et al., 2011). BIN2 negatively regulates BR signaling by phosphorylating and thereby promoting cytoplasmic retention of transcription factors, such as BRASSINAZOLE RESISTANT 1 (BZR1) and BRI1 EMS SUPPRESSOR1 (BES1), through interaction with 14-3-3 protein (Yang et al., 2011). Dephosphorylation of BZR1 and BES1 by protein phosphatase 2A, relieve cytoplasmic retention, allowing their nuclear translocation and binding to target promoters (Tang et al., 2011). Interestingly, the promoter of FLD contains one BR-responsive element (BRRE; Zhang et al., 2013b). Electrophoretic mobility shift assay (EMSA) shows that BRRE of FLOWERING LOCUS D promoter binds with the recombinant MBP-BZR1 protein but not the maltose binding protein (MBP; Zhang et al., 2013b). In addition, chromatin immuno-precipitation with GFP antibody shows enrichment of CFP-BZR1 in FLD promoter. The physical association and transcriptional analyses suggest that BZR1 binds to promoter of FLD and negatively regulates its expression (Zhang et al., 2013b). In immune signaling, FLAGELIN SENSING2 (FLS2) and ELONGATION FACTOR TU RECEPTOR (EFR), the pattern receptors for bacterial flagellin and elongation factor Tu respectively, heteromerize with BAK1 (Chinchilla et al., 2009; Roux et al., 2011; Sun et al., 2013). BAK1 phosphorylate BOTRYTIS-INDUCED KINASE1 (BIK1), a receptor-like cytoplasmic kinase that positively regulates plant immunity (Lu et al., 2010; Zhang et al., 2010). However, the BIK1 acts as a negative regulator of BR signaling. The *bik1* mutant plants show enhancement in dephosphorylation of BZR1 and BES1 (Lin et al., 2013), Thus activation of BIK1 by pathogens may inactivate BZR1 (through cytoplasmic retention) and thereby induce expression of FLD (**Figure 3**).

## Chromatin Remodeling

Eukaryotic DNA is packed into nucleosomes, which must transiently unpack during transcription. Alteration of nucleosome density, also known as chromatin remodeling, affects transcription of genes. In nucleosomes, DNA is wrapped around histone octamers consisting of two copies each of H2A, H2B, H3 and H4 (Kamakaka and Biggins, 2005). Post-translational modifications of histones as well as methylation of cytosine residues in DNA affect chromatin composition. The modifications of histones include methylation, acetylation, ubiquitination, and phosphorylation (Geiman and Robertson, 2002; Nowak and Corces, 2004). Usually, DNA methylation leads to suppression of transcriptional activity, whereas, acetylation of histones, especially in H3 and H4, activates transcription (Vaillant and Paszkowski, 2007). In contrast, methylation of histones can affect transcription both positively and negatively, depending on the histone protein and position of the modification (Zhang, 2008). Histone replacement, a process of substitution of canonical histones with histone variants, is also associated with chromatin remodeling (Kamakaka and Biggins, 2005; March-Diaz et al., 2008). Higher eukaryotes including plants possess machinery to initiate and maintain both DNA and histone modifications. Evidence suggests that both flowering and SAR are regulated by epigenetic modifications; interestingly, with machinery shared by both the pathways.

### Pathogen- and SA- Induced Histone Modification

Expression of several SA responsive genes is epigenetically regulated. Exogenous application of benzothiadiazole (BTH), a chemical analog of SA and potential SAR inducer, induces accumulation of modified histones that favor transcription, such as acetylated histone 3 (H3Ac), and di- and tri- methylated histone 3 at lysine 4 (H3K4me2 and H3K4me3) in the promoters of several *WRKY* genes, whose functions are associated with SAR activation (Jaskiewicz et al., 2011). Exogenous application of SA also induces such modifications in the *PR-1* promoter (Mosher et al., 2006). During SAR activation upon primary infection, the systemic tissues undergo similar epigenetic modifications, which is associated with robust expression of these defense related genes during challenge inoculation (Conrath, 2011; Singh et al., 2014b). Under stress-free condition, SUPPRESSOR OF PR-1 INDUCIBLE 1 (SNI1), a negative regulator of SAR, is thought to contribute to maintaining the basal expression of *PR-1* and *WRKY* genes by reducing these histones marks (Mosher et al., 2006).

### Histone Modifications; Flowering and SAR

*FLD* codes for an *Arabidopsis* ortholog of human LYSINE SPECIFIC DEMETHYLASE 1 (LSD1; Liu et al., 2007). FLD, an approximately 96 KDa protein contains a small DNA binding SWRIM domain, and a large polyamine oxidase (PAO) domain (He et al., 2003). Transcriptional co-repressor complexes containing PAO domains are one of the major regulators of gene expression in animals (Jepsen and Rosenfeld, 2002). LSD1 is a component of a co-repressor complex, with histone demethylase activity (Shi et al., 2004). The biochemical function of FLD has not been ascertained. However, the *fld* loss-of-function mutants show increased occupancy of methylated H3K4 in *FLC* locus as might be expected based on its structural similarity to LSD1, which is a histone demethylase (Liu et al., 2007; Singh et al., 2014b). In addition, the FLC locus also shows increased accumulation of acetylated H3 in the *fld* mutant background, both of which support the observation of increased expression of *FLC* in *fld* mutants (He et al., 2003; Singh et al., 2014b). However, in contrast to the *FLC* locus, promoters of *WRKY6* and *WRKY29* genes show reduced accumulation of methylated H3K4 and acetylated H3 (Singh et al., 2014b). Nevertheless, experiments suggest that the histone demethylase activity of FLD is important for SAR activation and flowering. For example, exogenous application of histone demethylase inhibitor *trans*-2-phenylcyclopropylamine (2-PCPA) results in an *fld* loss-of-function phenotype in terms of both flowering and SAR activation (Singh et al., 2014a). Application of 2-PCPA results in impairment of SAR activation, suppression of accumulation of methylated H3K4 in WRKY promoters and delays flowering (Singh et al., 2014a). FLD targets for SAR induction remain unidentified. It is postulated that the effect of FLD on histone modification of WRKY genes is indirect and may be mediated through other factors, functions of which are modulated by FLD (Singh et al., 2013, 2014a,b).

Histone deacetylases (HDACs) are often found in multiprotein co-repressor complexes. HISTONE DEACETYLASE 19 (HDA19) of *Arabidopsis* is a yeast REDUCED POTASSIUM DEFICIENCY 3 (RPD3)-like protein that affects both flowering and SAR (Zhou et al., 2005; Choi et al., 2012; Krogan et al., 2012; Wang et al., 2014). HDA19 interacts with LEUNIG/SEUSS co-repressor complex and negatively regulates expression of the floral patterning gene AGAMOUS (AG; Gonzalez et al., 2007). The *hda*19 mutant accumulates SA and has increased expression of SA-inducible genes such as *EDS1*, *PAD4*, *ICS1* as well as *PR* genes, providing resistance against *P. syringae* (Choi et al., 2012). HDA19, a putative corepressor has been found to directly associate with and deacetylate histones at the *PR-1* and *PR-2* promoters and repress their expression by modifying histones (Choi et al., 2012).

### Histone Replacement in SAR and Flowering

The mutants that constitutively activate SAR, show disease resistance, accumulation of SA and expression of PR genes without pathogen challenge, and also often develop microscopic cell death (Jirage et al., 2001; Nandi et al., 2005; Swain et al., 2011). Substitution of canonical histone H2A with H2A.Z variant is a mechanism of chromatin remodeling that is associated with early flowering and activation of constitutive SAR. Replacement of histone H2A with H2A.Z requires a multi-subunit complex, SWI2/SNF2-RELATED 1 (SWR1) in yeast and SNF2-RELATED CBP ACTIVATOR PROTEIN (SRCAP) in humans (Krogan et al., 2003; Kobor et al., 2004; Mizuguchi et al., 2004). *Arabidopsis* proteins PHOTOPERIOD-INDEPENDENT EARLY FLOWERING 1 (PIE1), ACTIN-RELATED PROTEIN 6 and SERRATED LEAVES AND EARLY FLOWERING (SEF) are related to SWR1 and SRCAP protein complex components (Noh and Amasino, 2003; March-Diaz et al., 2007, 2008). The PIE/ARP6/SEF complex is functional equivalent of yeast SWRI1 complex, components of which are required for deposition of H2A.Z variant in genes like FLC that negatively regulates flowering (Martin-Trillo et al., 2006; Deal et al., 2007; March-Diaz et al., 2007). Mutations in these genes result in down regulation of FLC and early flowering (**Figure 3**). Mutations in *Arabidopsis* *HTA9* and *HTA11* genes that code for H2A.Z also result in developmental abnormalities and early flowering (**Figure 3**), very similar to the mutants of PEI/ARP6/SEF complex (March-Diaz et al., 2008). The double mutant *hta9hta11* shows reduction in *FLC* expression and concomitant induction of *FT* expression similar to *sef* and *pie1* mutants (Amasino, 1996). As an interesting correlation between SAR and flowering, the *pie1*, *sef,* and *hta9hta11* mutants show activation of constitutive SAR (March-Diaz et al., 2008). Consequently, the *pie1*, *sef,* and *hta9hta11* mutants show spontaneous cell death, and support less bacterial growth than wild-type plants (March-Diaz et al., 2008). The *pie1* mutants also show constitute activation of *PR1*, *WRKY38* and *WRKY18*, expression of which are associated with SAR activation (Wang et al., 2006).

## Sumoylation Regulators Connect SAR and Flowering

Ubiquitin and SMALL UBIQUITIN-LIKE MODIFIER (SUMO) attach to a wide range of proteins, and alter their function and longevity in cells (Miura et al., 2007). SUMO covalently attaches to lysine residues of target proteins through E3 SUMO ligase (Wilkinson and Henley, 2010). SUMO conjugation modifies the conformation of target proteins and influences their interaction with other proteins (Hickey et al., 2012). SUMO modifications have been implicated in many biological processes including, nutrition metabolism, abiotic stress response, flowering, and immunity (Miura et al., 2005; Catala et al., 2007; Lee et al., 2007a; Zhang et al., 2013a). *Arabidopsis* SIZ1 (AtSIZ1) is an ortholog of mammalian and yeast E3 SUMO ligase (Miura et al., 2005). AtSIZ1 negatively regulates SAR activation (Lee et al., 2007a). The mutants of *AtSIZ1*, show enhanced expression of PHYTOALEXIN DEFICIENT 4 (PAD4) and ENHANCED DISEASE SUSCEPTIBILITY 1 (EDS1) that positively regulate SA biosynthesis. The *atsiz1* mutants also accumulate SA and SA-glucoside conjugates (SAG), express SAR marker *PR-1* constitutively, and are resistant to pathogens. All these phenotypes of *atsiz1* are dependent on SA accumulation, as *NahG* expression in the *atsiz1* mutant background, abolishes all SAR-associated responses (Lee et al., 2007a). As a very interesting cross-connection between SAR and flowering, it has been reported that AtSIZ1 promotes FLC expression and thereby negatively regulates the flowering transition (**Figure 3**; Jin et al., 2008). Very recently, it has also been shown that AtSIZ1 physically interacts with FLC and influences its stability (**Figure 3**; Son et al., 2014).

Reversal of SUMO conjugation is carried out by SUMO protease. The *early in short days 4* (*esd4*) mutant has a mutation in the SUMO protease and thus accumulates SUMO conjugates (Villajuana-Bonequi et al., 2014). Though, the exact cause of early flowering in *esd4* mutant is not known, it is believed that physiological stress caused by hyper-accumulation of SUMO conjugates may result in early flowering. Interestingly, a genetic screen for suppressors of *esd4*, identified a mutant of the SA biosynthetic gene *isochorismate synthase 1* (*ICS1*/*SID2*; Villajuana-Bonequi et al., 2014). Early flowering of *esd4* is partially rescued by the reduced SA levels in the *esd4 sid2* double mutant (Villajuana-Bonequi et al., 2014). Thus early flowering in *esd4* mutant is also associated with SA signaling activation, an integral event of SAR activation.

## Reverse Association between Flowering and SAR

The studies described above, provided evidence to support the idea that the flowering and SAR signaling pathways are highly integrated. However, there are reports that contradict this idea. For example, the *HOPW1-1-INTERACTING3* (*WIN3; alias PBS3, GDG1, GH3.12*) gene of *Arabidopsis* is a positive regulator for SAR and negative regulator of flowering (Wang et al., 2011). The *WIN3* gene product codes for an enzyme that conjugates aminoacids to 4-aminobenzoate or 4-hydroxybenzoate; a process which is required for SA biosynthesis (Okrent et al., 2009). WIN3 expression is induced by SA, and its function is needed for pathogenesis associated SA responses (Nobuta et al., 2007; Wang et al., 2011). However, mutation in *WIN3* results in early flowering under long-day conditions (Lee et al., 2007b; Wang et al., 2011). The reverse-association is also observed in plants undergoing the shade avoidance response. Since plants absorb more red than far-red light, the red:far-red ratio gets reduced under dense vegetation. The reduced red:far-red ratio enhances early flowering in *Arabidopsis*, a component of the shade-avoidance response (Halliday et al., 1994, 2003). In contrast, plants grown in low red:far-red ratio exhibit compromised defense response in the form of reduced SA dependent PR1 and WRKY expression (de Wit et al., 2013). The lack of an association between SAR and flowering is observed in mutants such as *npr1* and *sfd1*, which are defective in SAR but not in flowering. Therefore, these reports indicate that the SAR and flowering pathways are genetically separable, even though they share common inducers, such as SA.

## Conclusion

It appears that some of the molecular machinery that regulates flowering time is shared by the SAR activation processes in plants. A number of studies suggest that retention of infection memory in plants is mostly mediated through epigenetic mechanisms. While generating resistance against invading pathogens, plant tissues generate signals that are capable of long distance transport to carry infection information to distant tissues. Upon arrival in distal tissues, mobile signals are perceived, leading to biosynthesis and accumulation of SA, which is required for SAR activation as well as the floral transition. In addition, the perception of mobile signals also initiates epigenetic modification of certain key genes which contribute to infection memory development and SAR associated priming defense responses. Alteration of histone methylation and acetylation, and histone replacement influence flowering and SAR. Suppression of SAR in healthy plants and flowering during vegetative growth, are highly important for overall growth, development and productivity of plants. Emerging data strongly suggest common genetic and epigenetic regulators for flowering and SAR.

## Conflict of Interest Statement

The authors declare that the research was conducted in the absence of any commercial or financial relationships that could be construed as a potential conflict of interest.

## Author contributions

Both the authors equally contributed to the manuscript.

## References

[B1] Amasino R. M. (1996). Control of flowering time in plants. *Curr. Opin. Genet. Dev.* 6 480–487 10.1016/S0959-437X(96)80071-2 8791538

[B2] Aukerman M. J. Amasino R. M. (1996). Molecular genetic analysis of flowering time in *Arabidopsis*. *Semin. Cell Dev. Biol.* 7 427–433 10.1006/scdb.1996.0053

[B3] Ballare C. L. Mazza C. A. Austin A. T. Pierik R. (2012). Canopy light and plant health. *Plant Physiol.* 160 145–155 10.1104/pp.112.200733 22802612 PMC3440192

[B4] Caruso F. L. Kuc J. (1977). Protection of watermelon and muskmelon against *Colletotrichum lagenarium* by *Colletotrichum lagenarium*. *Phytopathology* 67 1285–1289 10.1094/Phyto-67-1285

[B5] Catala R. Ouyang J. Abreu I. A. Hu Y. Seo H. Zhang X. (2007). The *Arabidopsis* E3 SUMO ligase SIZ1 regulates plant growth and drought responses. *Plant Cell* 19 2952–2966 10.1105/tpc.106.049981 17905899 PMC2048692

[B6] Chailakhyan M. K. (1936). New facts in support of the hormonal theory of plant development. *C. R. Acad. Sci. URSS* 13 79–83.

[B7] Champigny M. J. Isaacs M. Carella P. Faubert J. Fobert P. R. Cameron R. K. (2013). Long distance movement of DIR1 and investigation of the role of DIR1-like during systemic acquired resistance in *Arabidopsis*. *Front. Plant. Sci.* 4:230 10.3389/fpls.2013.00230 PMC3701462 23847635

[B8] Chanda B. Xia Y. Mandal M. K. Yu K. Sekine K. T. Gao Q. M. (2011). Glycerol-3-phosphate is a critical mobile inducer of systemic immunity in plants. *Nat. Genet.* 43 421–427 10.1038/ng.798 21441932

[B9] Chandra-Shekara A. C. Gupte M. Navarre D. Raina S. Raina R. Klessig D. (2006). Light-dependent hypersensitive response and resistance signaling against turnip crinkle virus in *Arabidopsis*. *Plant J.* 45 320–334 10.1111/j.1365-313X.2005.02618.x 16412080

[B10] Chaturvedi R. Krothapalli K. Makandar R. Nandi A. Sparks A. A. Roth M. R. (2008). Plastid omega3-fatty acid desaturase-dependent accumulation of a systemic acquired resistance inducing activity in petiole exudates of *Arabidopsis thaliana* is independent of jasmonic acid. *Plant J.* 54 106–117 10.1111/j.1365-313X.2007.03400.x 18088304

[B11] Chaturvedi R. Venables B. Petros R. A. Nalam V. Li M. Wang X. (2012). An abietane diterpenoid is a potent activator of systemic acquired resistance. *Plant J.* 71 161–172 10.1111/j.1365-313X.2012.04981.x 22385469

[B12] Chinchilla D. Shan L. He P. De Vries S. Kemmerling B. (2009). One for all: the receptor-associated kinase BAK1. *Trends Plant Sci.* 14 535–541 10.1016/j.tplants.2009.08.002 19748302 PMC4391746

[B13] Choi S. M. Song H. R. Han S. K. Han M. Kim C. Y. Park J. (2012). HDA19 is required for the repression of salicylic acid biosynthesis and salicylic acid-mediated defense responses in *Arabidopsis*. *Plant J.* 71 135–146 10.1111/j.1365-313X.2012.04977.x 22381007

[B14] Cleland C. F. Ajami A. (1974). Identification of the flower-inducing factor isolated from aphid honeydew as being salicylic acid. *Plant Physiol.* 54 904–906 10.1104/pp.54.6.904 16658997 PMC366630

[B15] Clouse S. D. (2011). Brassinosteroid signal transduction: from receptor kinase activation to transcriptional networks regulating plant development. *Plant Cell* 23 1219-1230. 10.1105/tpc.111.084475 PMC3101532 21505068

[B16] Conrath U. (2011). Molecular aspects of defence priming. *Trends Plant Sci.* 16 524–531 10.1016/j.tplants.2011.06.004 21782492

[B17] Corbesier L. Vincent C. Jang S. Fornara F. Fan Q. Searle I. (2007). FT protein movement contributes to long-distance signaling in floral induction of *Arabidopsis*. *Science* 316 1030–1033 10.1126/science.1141752 17446353

[B18] Deal R. B. Topp C. N. Mckinney E. C. Meagher R. B. (2007). Repression of flowering in *Arabidopsis* requires activation of FLOWERING LOCUS C expression by the histone variant H2A.Z*. Plant Cell* 19 74–83 10.1105/tpc.106.048447 PMC1820970 17220196

[B19] Dempsey D. A. Klessig D. F. (2012). SOS - too many signals for systemic acquired resistance? *Trends Plant Sci*. 17 538–545 10.1016/j.tplants.2012.05.011 22749315

[B20] de Wit M. Spoel S. H. Sanchez-Perez G. F. Gommers C. M. Pieterse C. M. Voesenek L. A. (2013). Perception of low red:far-red ratio compromises both salicylic acid- and jasmonic acid-dependent pathogen defences in *Arabidopsis*. *Plant J.* 75 90–103 10.1111/tpj.12203 23578319

[B21] Dong X. (2004). NPR 1 all things considered. *Curr. Opin. Plant Biol.* 7 547–552 10.1016/j.pbi.2004.07.005 15337097

[B22] Durrant W. E. Dong X. (2004). Systemic acquired resistance. *Annu. Rev. Phytopathol.* 42 185–209 10.1146/annurev.phyto.42.040803.140421 15283665

[B23] Eulgem T. Tsuchiya T. Wang X. J. Beasley B. Cuzick A. Tor M. (2007). EDM2 is required for RPP7-dependent disease resistance in *Arabidopsis* and affects RPP7 transcript levels. *Plant J.* 49 829–839 10.1111/j.1365-313X.2006.02999.x 17253987

[B24] Fragniere C. Serrano M. Abou-Mansour E. Metraux J. P. L’haridon F. (2011). Salicylic acid and its location in response to biotic and abiotic stress. *FEBS Lett.* 585 1847–1852 10.1016/j.febslet.2011.04.039 21530511

[B25] Fu Z. Q. Dong X. (2013). Systemic acquired resistance: turning local infection into global defense. *Annu. Rev. Plant Biol.* 64 839–863 10.1146/annurev-arplant-042811-105606 23373699

[B26] Fujioka S. Sakurai A. Yamaguchi I. Murofushi N. Takahashi N. Kaihara S. (1987). Isolation and identification of L-Pipecolic acid and nicotinamide as flower-inducing substances in *Lemna*. *Plant Cell Physiol.* 28 995–1003.

[B27] Gaffney T. Friedrich L. Vernooij B. Negrotto D. Nye G. Uknes S. (1993). Requirement of salicylic acid for the induction of systemic acquired resistance. *Science* 261 754–756 10.1126/science.261.5122.754 17757215

[B28] Geiman T. M. Robertson K. D. (2002). Chromatin remodeling, histone modifications, and DNA methylation-how does it all fit together? *J. Cell Biochem.* 87 117–125 10.1002/jcb.10286 12244565

[B29] Gonzalez D. Bowen A. J. Carroll T. S. Conlan R. S. (2007). The transcription corepressor LEUNIG interacts with the histone deacetylase HDA19 and mediator components MED14 (SWP) and CDK8 (HEN3) to repress transcription. *Mol. Cell. Biol.* 27 5306–5315 10.1128/MCB.01912-1916 17526732 PMC1952085

[B30] Griebel T. Zeier J. (2008). Light regulation and daytime dependency of inducible plant defenses in *Arabidopsis*: phytochrome signaling controls systemic acquired resistance rather than local defense. *Plant Physiol.* 147 790–801 10.1104/pp.108.119503 18434604 PMC2409012

[B31] Guedes M. E. M. Richmond S. Kuc J. (1980). Induced systemic resistance to anthracnose in cucumber as influenced by the location of the inducer inoculation with *Colletotrichum lagenarium* and the onset of flowering and fruiting physiol. *Plant Pathol.* 17 229–233.

[B32] Halliday K. J. Koornneef M. Whitelam G. C. (1994). Phytochrome B and at least one other phytochrome mediate the accelerated flowering response of *Arabidopsis thaliana* L. to low Red/Far-Red ratio. *Plant Physiol.* 104 1311–1315. 12232170 10.1104/pp.104.4.1311 PMC159295

[B33] Halliday K. J. Salter M. G. Thingnaes E. Whitelam G. C. (2003). Phytochrome control of flowering is temperature sensitive and correlates with expression of the floral integrator FT. *Plant J.* 33 875–885 10.1046/j.1365-313X.2003.01674.x 12609029

[B34] He Y. Amasino R. M. (2005). Role of chromatin modification in flowering-time control. *Trends Plant Sci.* 10 30–35 10.1016/j.tplants.2004.11.003 15642521

[B35] He Y. H. Michaels S. D. Amasino R. M. (2003). Regulation of flowering time by histone acetylation in *Arabidopsis*. *Science* 302 1751–1754 10.1126/science.1091109 14593187

[B36] Helliwell C. A. Wood C. C. Robertson M. James Peacock W. Dennis E. S. (2006). The *Arabidopsis* FLC protein interacts directly in vivo with SOC1 and FT chromatin and is part of a high-molecular-weight protein complex. *Plant J.* 46 183–192 10.1111/j.1365-313X.2006.02686.x 16623882

[B37] Henderson I. R. Dean C. (2004). Control of *Arabidopsis* flowering: the chill before the bloom. *Development* 131 3829–3838 10.1242/dev.01294 15289433

[B38] Hickey C. M. Wilson N. R. Hochstrasser M. (2012). Function and regulation of SUMO proteases. *Nat. Rev. Mol. Cell Biol.* 13 755–766 10.1038/nrm3478 23175280 PMC3668692

[B39] Iriti M. Faoro F. (2007). Review of innate and specific immunity in plants and animals. *Mycopathologia* 164 57–64 10.1007/s11046-007-9026-9027 17554637

[B40] Islam S. Z. Babadoost M. Bekal S. Lambert K. (2008). Red Light-induced systemic disease resistance against Root-knot nematode *Meloidogyne javanica* and *Pseudomonas syringae* pv. tomato DC 3000. *J. Phytopathol*. 156 708–714 10.1111/j.1439-0434.2008.01435.x

[B41] Jaskiewicz M. Conrath U. Peterhansel C. (2011). Chromatin modification acts as a memory for systemic acquired resistance in the plant stress response. *EMBO Rep.* 12 50–55 10.1038/embor.2010.186 21132017 PMC3024125

[B42] Jepsen K. Rosenfeld M. G. (2002). Biological roles and mechanistic actions of co-repressor complexes. *J. Cell Sci.* 115 689–698. 11865025 10.1242/jcs.115.4.689

[B43] Jin J. B. Jin Y. H. Lee J. Miura K. Yoo C. Y. Kim W. Y. (2008). The SUMO E3 ligase, AtSIZ 1 regulates flowering by controlling a salicylic acid-mediated floral promotion pathway and through affects on FLC chromatin structure. *Plant J.* 53 530–540 10.1111/j.1365-313X.2007.03359.x 18069938 PMC2254019

[B44] Jirage D. Zhou N. Cooper B. Clarke J. D. Dong X. Glazebrook J. (2001). Constitutive salicylic acid-dependent signaling in cpr1 and cpr6 mutants requires PAD4. *Plant J.* 26 395–407 10.1046/j.1365-313X.2001.2641040.x 11439127

[B45] Jones J. D. Dangl J. L. (2006). The plant immune system. *Nature* 444 323–329 10.1038/nature05286 17108957

[B46] Jung H. W. Tschaplinski T. J. Wang L. Glazebrook J. Greenberg J. T. (2009). Priming in systemic plant immunity. *Science* 324 89–91 10.1126/science.1170025 19342588

[B47] Kamakaka R. T. Biggins S. (2005). Histone variants: deviants? *Genes Dev.* 19 295–310 10.1101/gad.1272805 15687254

[B48] Kangasjarvi S. Neukermans J. Li S. Aro E. M. Noctor G. (2012). Photosynthesis, photorespiration, and light signalling in defence responses. *J. Exp. Bot.* 63 1619–1636 10.1093/jxb/err402 22282535

[B49] Khurana J. P. Cleland C. F. (1992). Role of salicylic acid and benzoic acid in flowering of a Photoperiod-Insensitive strain, *Lemna paucicostata* LP6. *Plant Physiol.* 100 1541–1546 10.1104/pp.100.3.1541 16653155 PMC1075817

[B50] Kim T. W. Guan S. Burlingame A. L. Wang Z. Y. (2011). The CDG1 kinase mediates brassinosteroid signal transduction from BRI1 receptor kinase to BSU1 phosphatase and GSK3-like kinase BIN2. *Mol. Cell* 43 561–571 10.1016/j.molcel.2011.05.037 21855796 PMC3206214

[B51] Kinkema M. Fan W. Dong X. (2000). Nuclear localization of NPR1 is required for activation of PR gene expression. *Plant Cell* 12 2339–2350 10.1105/tpc.12.12.2339 11148282 PMC102222

[B52] Knott J. E. (1934). Effect of localized photoperiod on spinach. *Proc. Am. Soc. Hortic. Sci.* 31 152–154.

[B53] Kobor M. S. Venkatasubrahmanyam S. Meneghini M. D. Gin J. W. Jennings J. L. Link A. J. (2004). A protein complex containing the conserved Swi2/Snf2-related ATPase Swr1p deposits histone variant H2A.Z into euchromatin. *PLoS Biol.* 2:E131. 10.1371/journal.pbio.0020131 PMC374244 15045029

[B54] Krogan N. J. Keogh M. C. Datta N. Sawa C. Ryan O. W. Ding H. (2003). A Snf2 family ATPase complex required for recruitment of the histone H2A variant Htz1. *Mol. Cell* 12 1565–1576 10.1016/S1097-2765(03)00497-0 14690608

[B55] Krogan N. T. Hogan K. Long J. A. (2012). APETALA2 negatively regulates multiple floral organ identity genes in *Arabidopsis* by recruiting the co-repressor TOPLESS and the histone deacetylase HDA19. *Development* 139 4180–4190 10.1242/dev.085407dev.085407 23034631 PMC3478687

[B56] Kuc J. Richmond S. (1977). Aspects of protection of cucumber against *Colletotrichum lagenarium* by *Colletotrichum lagenarium*. *Phytopathology* 67 533–536 10.1094/Phyto-67-533

[B57] Lawton K. A. Friedrich L. Hunt M. Weymann K. Delaney T. Kessmann H. (1996). Benzothiadiazole induces disease resistance in *Arabidopsis* by activation of the systemic acquired resistance signal transduction pathway. *Plant J.* 10 71–82 10.1046/j.1365-313X.1996.10010071.x 8758979

[B58] Lee J. Nam J. Park H. C. Na G. Miura K. Jin J. B. (2007a). Salicylic acid-mediated innate immunity in *Arabidopsis* is regulated by SIZ1 SUMO E3 ligase. *Plant J.* 49 79–90 10.1111/j.1365-313X.2006.02947.x 17163880

[B59] Lee M. W. Lu H. Jung H. W. Greenberg J. T. (2007b). A key role for the *Arabidopsis* WIN3 protein in disease resistance triggered by *Pseudomonas syringae* that secrete AvrRpt2. *Mol. Plant Microbe Interact.* 20 1192–1200 10.1094/MPMI-20-10-1192 17918621

[B60] Li C. Luo L. Fu Q. Niu L. Xu Z. F. (2014). Isolation and functional characterization of JcFT, a FLOWERING LOCUS T (FT) homologous gene from the biofuel plant *Jatropha curcas*. *BMC Plant Biol.* 14:125 10.1186/1471-2229-14-125 PMC4036407 24886195

[B61] Lin W. Lu D. Gao X. Jiang S. Ma X. Wang Z. (2013). Inverse modulation of plant immune and brassinosteroid signaling pathways by the receptor-like cytoplasmic kinase BIK1. *Proc. Natl. Acad. Sci. U.S.A.* 110 12114–12119 10.1073/pnas.1302154110 23818580 PMC3718091

[B62] Liu F. Quesada V. Crevillen P. Baurle I. Swiezewski S. Dean C. (2007). The *Arabidopsis* RNA-binding protein FCA requires a lysine-specific demethylase 1 homolog to downregulate FLC. *Mol. Cell* 28 398–407 10.1016/j.molcel.2007.10.018 17996704

[B63] Lu D. Wu S. Gao X. Zhang Y. Shan L. He P. (2010). A receptor-like cytoplasmic kinase, BIK 1 associates with a flagellin receptor complex to initiate plant innate immunity. *Proc. Natl. Acad. Sci. U.S.A.* 107 496–501 10.1073/pnas.0909705107 20018686 PMC2806711

[B64] Luna E. Bruce T. J. Roberts M. R. Flors V. Ton J. (2012). Next-generation systemic acquired resistance. *Plant Physiol.* 158 844–853 10.1104/pp.111.187468 22147520 PMC3271772

[B65] March-Diaz R. Garcia-Dominguez M. Florencio F. J. Reyes J. C. (2007). SEF, a new protein required for flowering repression in *Arabidopsis*, interacts with PIE1 and ARP6. *Plant Physiol.* 143 893–901 10.1104/pp.106.092270 17142478 PMC1803727

[B66] March-Diaz R. Garcia-Dominguez M. Lozano-Juste J. Leon J. Florencio F. J. Reyes J. C. (2008). Histone H2A.Z and homologues of components of the SWR1 complex are required to control immunity in *Arabidopsis*. *Plant J.* 53 475–487 10.1111/j.1365-313X.2007.03361.x 17988222

[B67] Martin-Trillo M. Lázaro A. Poethig R. S. Gómez-Mena C. Piñeiro M. A. Martinez-Zapater J. M. (2006). EARLY IN SHORT DAYS 1 (ESD1) encodes ACTIN-RELATED PROTEIN 6 (AtARP6), a putative component of chromatin remodelling complexes that positively regulates FLC accumulation in *Arabidopsis*. *Development* 133 1241–1252 10.1242/dev.02301 16495307

[B68] Martinez C. Pons E. Prats G. Leon J. (2004). Salicylic acid regulates flowering time and links defence responses and reproductive development. *Plant J.* 37 209–217 10.1046/j.1365-313X.2003.01954.x 14690505

[B69] Metraux J. P. Signer H. Ryals J. Ward E. Wyss-Benz M. Gaudin J. (1990). Increase in salicylic acid at the onset of systemic acquired resistance in cucumber. *Science* 250 1004–1006 10.1126/science.250.4983.1004 17746926

[B70] Michaels S. D. Amasino R. M. (1999). FLOWERING LOCUS C encodes a novel MADS domain protein that acts as a repressor of flowering. *Plant Cell* 11 949–956 10.1105/tpc.11.5.949 10330478 PMC144226

[B71] Miura K. Jin J. B. Hasegawa P. M. (2007). Sumoylation, a post-translational regulatory process in plants. *Curr. Opin. Plant Biol.* 10 495–502 10.1016/j.pbi.2007.07.002 17720613

[B72] Miura K. Rus A. Sharkhuu A. Yokoi S. Karthikeyan A. S. Raghothama K. G. (2005). The *Arabidopsis* SUMO E3 ligase SIZ1 controls phosphate deficiency responses. *Proc. Natl. Acad. Sci. U.S.A.* 102 7760–7765 10.1073/pnas.0500778102 15894620 PMC1140425

[B73] Mizuguchi G. Shen X. Landry J. Wu W. H. Sen S. Wu C. (2004). ATP-driven exchange of histone H2AZ variant catalyzed by SWR1 chromatin remodeling complex. *Science* 303 343–348 10.1126/science.1090701 14645854

[B74] Mosher R. A. Durrant W. E. Wang D. Song J. Dong X. (2006). A comprehensive structure-function analysis of *Arabidopsis* SNI1 defines essential regions and transcriptional repressor activity. *Plant Cell* 18 1750–1765 10.1105/tpc.105.039677 16766691 PMC1488919

[B75] Nandi A. Moeder W. Kachroo P. Klessig D. F. Shah J. (2005). Arabidopsis ssi2-conferred susceptibility to *Botrytis cinerea* is dependent on EDS5 and PAD4. *Mol. Plant Microbe Interact.* 18 363–370 10.1094/MPMI-18-0363 15828688

[B76] Nandi A. Welti R. Shah J. (2004). The *Arabidopsis thaliana* dihydroxyacetone phosphate reductase gene SUPPRESSSOR OF FATTY ACID DESATURASE DEFICIENCY1 is required for glycerolipid metabolism and for the activation of systemic acquired resistance. *Plant Cell* 16 465–477 10.1105/tpc.016907 14729910 PMC341917

[B77] Navarova H. Bernsdorff F. Doring A. C. Zeier J. (2012). Pipecolic acid, an endogenous mediator of defense amplification and priming, is a critical regulator of inducible plant immunity. *Plant Cell* 24 5123–5141 10.1105/tpc.112.103564 23221596 PMC3556979

[B78] Nimchuk Z. Eulgem T. Holt B. F. III Dangl J. L. (2003). Recognition and response in the plant immune system. *Annu. Rev. Genet.* 37 579–609 10.1146/annurev.genet.37.110801.142628 14616074

[B79] Nobuta K. Okrent R. A. Stoutemyer M. Rodibaugh N. Kempema L. Wildermuth M. C. (2007). The GH3 acyl adenylase family member PBS3 regulates salicylic acid-dependent defense responses in *Arabidopsis*. *Plant Physiol.* 144 1144–1156 10.1104/pp.107.097691 17468220 PMC1914169

[B80] Noh Y. S. Amasino R. M. (2003). PIE 1 an ISWI family gene, is required for FLC activation and floral repression in *Arabidopsis*. *Plant Cell* 15 1671–1682 10.1105/tpc.012161 12837955 PMC165409

[B81] Nowak S. J. Corces V. G. (2004). Phosphorylation of histone H3: a balancing act between chromosome condensation and transcriptional activation. *Trends Genet.* 20 214–220 10.1016/j.tig.2004.02.007 15041176

[B82] Okrent R. A. Brooks M. D. Wildermuth M. C. (2009). Arabidopsis GH3.12 (PBS3) conjugates amino acids to 4-substituted benzoates and is inhibited by salicylate. *J. Biol. Chem.* 284 9742–9754 10.1074/jbc.M806662200 19189963 PMC2665095

[B83] Ross A. F. (1961). Systemic acquired resistance induced by localized virus infections in plants. *Virology* 14 340–358 10.1016/0042-6822(61)90319-1 13743578

[B84] Roux M. Schwessinger B. Albrecht C. Chinchilla D. Jones A. Holton N. (2011). The *Arabidopsis* leucine-rich repeat receptor-like kinases BAK1/SERK3 and BKK1/SERK4 are required for innate immunity to hemibiotrophic and biotrophic pathogens. *Plant Cell* 23 2440–2455 10.1105/tpc.111.084301 21693696 PMC3160018

[B85] Ryals J. A. Neuenschwander U. H. Willits M. G. Molina A. Steiner H. Y. Hunt M. D. (1996). Systemic acquired resistance. *Plant Cell* 8 1809–1819 10.1105/tpc.8.10.1809 12239363 PMC161316

[B86] Sano S. Aoyama M. Nakai K. Shimotani K. Yamasaki K. Sato M. H. (2014). Light-dependent expression of flg22-induced defense genes in *Arabidopsis*. *Front. Plant Sci.* 5:531 10.3389/fpls.2014.00531 PMC4191550 25346742

[B87] Schwessinger B. Ronald P. C. (2012). Plant innate immunity: perception of conserved microbial signatures. *Annu. Rev. Plant Biol.* 63 451–482 10.1146/annurev-arplant-042811-105518 22404464

[B88] Shi Y. Lan F. Matson C. Mulligan P. Whetstine J. R. Cole P. A. (2004). Histone demethylation mediated by the nuclear amine oxidase homolog LSD1. *Cell* 119 941–953 10.1016/j.cell.2004.12.012 15620353

[B89] Simpson G. G. Dean C. (2002). Arabidopsis, the rosetta stone of flowering time? *Science* 296 285–289 10.1126/science.296.5566.285296/5566/285 11951029

[B90] Simpson G. G. Gendall A. R. Dean C. (1999). When to switch to flowering. *Annu. Rev. Cell Dev. Biol.* 15 519–550 10.1146/annurev.cellbio.15.1.519 10611971

[B91] Singh V. Banday Z. Z. Nandi A. K. (2014a). Exogenous application of histone demethylase inhibitor trans-2-phenylcyclopropylamine mimics FLD loss-of-function phenotype in terms of systemic acquired resistance in *Arabidopsis thaliana*. *Plant Signal. Behav.* 10.4161/psb.29658 [Epub ahead of print]. PMC4203637 25763701

[B92] Singh V. Roy S. Singh D. Nandi A. K. (2014b). Arabidopsis flowering locus D influences systemic-acquired-resistance- induced expression and histone modifications of WRKY genes. *J. Biosci.* 39 119–126 10.1007/s12038-013-9407-7 24499796

[B93] Singh V. Roy S. Giri M. K. Chaturvedi R. Chowdhury Z. Shah J. (2013). Arabidopsis *thaliana* FLOWERING LOCUS D is required for systemic acquired resistance. *Mol. Plant Microbe Interact.* 26 1079–1088 10.1094/MPMI-04-13-0096-R 23745676

[B94] Slaughter A. Daniel X. Flors V. Luna E. Hohn B. Mauch-Mani B. (2012). Descendants of primed *Arabidopsis* plants exhibit resistance to biotic stress. *Plant Physiol.* 158 835–843 10.1104/pp.111.191593pp.111.191593 22209872 PMC3271771

[B95] Smith H. (2000). Phytochromes and light signal perception by plants–an emerging synthesis. *Nature* 407 585–591 10.1038/35036500 11034200

[B96] Son G. H. Park B. S. Song J. T. Seo H. S. (2014). FLC-mediated flowering repression is positively regulated by sumoylation. *J. Exp. Bot.* 65 339–351 10.1093/jxb/ert383ert383 24218331 PMC3883301

[B97] Sticher L. Mauch-Mani B. Metraux J. P. (1997). Systemic acquired resistance. *Annu. Rev. Phytopathol.* 35 235–270 10.1146/annurev.phyto.35.1.235 15012523

[B98] Suarez-Lopez P. Wheatley K. Robson F. Onouchi H. Valverde F. Coupland G. (2001). CONSTANS mediates between the circadian clock and the control of flowering in *Arabidopsis*. *Nature* 410 1116–1120 10.1038/35074138 11323677

[B99] Sun Y. Li L. Macho A. P. Han Z. Hu Z. Zipfel C. (2013). Structural basis for flg22-induced activation of the *Arabidopsis* FLS2-BAK1 immune complex. *Science* 342 624–628 10.1126/science.1243825 24114786

[B100] Swain S. Roy S. Shah J. Van Wees S. Pieterse C. M. Nandi A. K. (2011). Arabidopsis thaliana cdd1 mutant uncouples the constitutive activation of salicylic acid signalling from growth defects. *Mol. Plant Pathol.* 12 855–865 10.1111/j.1364-3703.2011.00717.x 21726384 PMC6640339

[B101] Tamaki S. Matsuo S. Wong H. L. Yokoi S. Shimamoto K. (2007). Hd3a protein is a mobile flowering signal in rice. *Science* 316 1033–1036 10.1126/science.1141753 17446351

[B102] Tang W. Kim T. W. Oses-Prieto J. A. Sun Y. Deng Z. Zhu S. (2008). BSKs mediate signal transduction from the receptor kinase BRI1 in *Arabidopsis*. *Science* 321 557–560 10.1126/science.1156973 18653891 PMC2730546

[B103] Tang W. Yuan M. Wang R. Yang Y. Wang C. Oses-Prieto J. A. (2011). PP2A activates brassinosteroid-responsive gene expression and plant growth by dephosphorylating BZR1. *Nat. Cell Biol.* 13 124–131 10.1038/ncb2151 21258370 PMC3077550

[B104] Tsuchiya T. Eulgem T. (2010). The *Arabidopsis* defense component EDM2 affects the floral transition in an FLC-dependent manner. *Plant J.* 62 518–528 10.1111/j.1365-313X.2010.04169.xTPJ4169 20149132

[B105] Tuzun S. Kuc J. (1985). Movement of a factor in tobacco infected with *Peronospora tabacina* Adam which systemically protect against blue mold. *Physiol. Plant Pathol.* 26 321–330 10.1016/0048-4059(85)90007-4

[B106] Vaillant I. Paszkowski J. (2007). Role of histone and DNA methylation in gene regulation. *Curr. Opin. Plant Biol.* 10 528–533 10.1016/j.pbi.2007.06.008 17692561

[B107] van Loon L. C. Rep M. Pieterse C. M. (2006). Significance of inducible defense-related proteins in infected plants. *Annu. Rev. Phytopathol.* 44 135–162 10.1146/annurev.phyto.44.070505.143425. 16602946

[B108] Varkonyi-Gasic E. Moss S. M. Voogd C. Wang T. Putterill J. Hellens R. P. (2013). Homologs of FT, CEN and FD respond to developmental and environmental signals affecting growth and flowering in the perennial vine kiwifruit. *New Phytol.* 198 732–746 10.1111/nph.12162 23577598

[B109] Verhage A. Van Wees S. C. Pieterse C. M. (2010). Plant immunity: it’s the hormones talking, but what do they say? *Plant Physiol.* 154 536–540 10.1104/pp.110.161570 20921180 PMC2949039

[B110] Villajuana-Bonequi M. Elrouby N. Nordstrom K. Griebel T. Bachmair A. Coupland G. (2014). Elevated salicylic acid levels conferred by increased expression of ISOCHORISMATE SYNTHASE 1 contribute to hyperaccumulation of SUMO1 conjugates in the *Arabidopsis* mutant early in short days 4. *Plant J.* 79 206–219 10.1111/tpj.12549 24816345

[B111] Vlot A. C. Klessig D. F. Park S. W. (2008). Systemic acquired resistance: the elusive signal(s). *Curr. Opin. Plant Biol.* 11 436–442 10.1016/j.pbi.2008.05.003 18614393

[B112] Wada K. C. Mizuuchi K. Koshio A. Kaneko K. Mitsui T. Takeno K. (2014). Stress enhances the gene expression and enzyme activity of phenylalanine ammonia-lyase and the endogenous content of salicylic acid to induce flowering in pharbitis. *J. Plant Physiol.* 171 895–902 10.1016/j.jplph.2014.03.008 24913046

[B113] Wang D. Amornsiripanitch N. Dong X. (2006). A genomic approach to identify regulatory nodes in the transcriptional network of systemic acquired resistance in plants. *PLoS Pathog.* 2:e123 10.1371/journal.ppat.0020123 PMC1635530 17096590

[B114] Wang G. F. Seabolt S. Hamdoun S. Ng G. Park J. Lu H. (2011). Multiple roles of WIN3 in regulating disease resistance, cell death, and flowering time in *Arabidopsis*. *Plant Physiol.* 156 1508–1519 10.1104/pp.111.176776 21543726 PMC3135961

[B115] Wang H. Jiang Y. P. Yu H. J. Xia X. J. Shi K. Zhou Y. H. (2010). Light quality affects incidence of powdery mildew, expression of defence-related genes and associated metabolism in cucumber plants. *Eur. J. Plant Pathol.* 127 125–135 10.1007/s10658-009-9577-9571

[B116] Wang Z. Cao H. Chen F. Liu Y. (2014). The roles of histone acetylation in seed performance and plant development. *Plant Physiol. Biochem.* 84C 125–133 10.1016/j.plaphy.2014.09.010 25270163

[B117] Ward E. R. Uknes S. J. Williams S. C. Dincher S. S. Wiederhold D. L. Alexander D. C. (1991). Coordinate gene activity in response to agents that induce systemic acquired resistance. *Plant Cell* 3 1085–1094 10.1105/tpc.3.10.1085 12324583 PMC160074

[B118] Wilkinson K. A. Henley J. M. (2010). Mechanisms, regulation and consequences of protein SUMOylation. *Biochem. J.* 428 133–145 10.1042/BJ20100158 20462400 PMC3310159

[B119] Wu Y. Zhang D. Chu J. Y. Boyle P. Wang Y. Brindle I. D. (2012). The *Arabidopsis* NPR1 protein is a receptor for the plant defense hormone salicylic acid. *Cell Rep.* 1 639–647 10.1016/j.celrep.2012.05.008 22813739

[B120] Yalpani N. Silverman P. Wilson T. M. Kleier D. A. Raskin I. (1991). Salicylic acid is a systemic signal and an inducer of pathogenesis-related proteins in virus-infected tobacco. *Plant Cell* 3 809–818 10.1105/tpc.3.8.809 1820820 PMC160048

[B121] Yang C. J. Zhang C. Lu Y. N. Jin J. Q. Wang X. L. (2011). The mechanisms of brassinosteroids’ action: from signal transduction to plant development. *Mol. Plant* 4 588–600 10.1093/mp/ssr020 21471332

[B122] Zeevaart J. A. (2006). Florigen coming of age after 70 years. *Plant Cell* 18 1783–1789 10.1105/tpc.106.043513 16905662 PMC1533981

[B123] Zeier J. Pink B. Mueller M. J. Berger S. (2004). Light conditions influence specific defence responses in incompatible plant-pathogen interactions: uncoupling systemic resistance from salicylic acid and PR-1 accumulation. *Planta* 219 673–683 10.1007/s00425-004-1272-z 15098125

[B124] Zhang J. Li W. Xiang T. Liu Z. Laluk K. Ding X. (2010). Receptor-like cytoplasmic kinases integrate signaling from multiple plant immune receptors and are targeted by a *Pseudomonas syringae* effector. *Cell Host Microbe* 7 290–301 10.1016/j.chom.2010.03.007 20413097

[B125] Zhang S. Qi Y. Liu M. Yang C. (2013a). SUMO E3 ligase AtMMS21 regulates drought tolerance in *Arabidopsis thaliana*(F). *J. Integr. Plant Biol.* 55 83–95 10.1111/jipb.12024 23231763

[B126] Zhang Y. Li B. Xu Y. Li H. Li S. Zhang D. (2013b). The cyclophilin CYP20-2 modulates the conformation of BRASSINAZOLE-RESISTANT 1 which binds the promoter of FLOWERING LOCUS D to regulate flowering in *Arabidopsis*. *Plant Cell* 25 2504–2521 10.1105/tpc.113.110296 23897924 PMC3753379

[B127] Zhang X. (2008). The epigenetic landscape of plants. *Science* 320 489–492 10.1126/science.1153996 18436779

[B128] Zhou C. Zhang L. Duan J. Miki B. Wu K. (2005). HISTONE DEACETYLASE19 is involved in jasmonic acid and ethylene signaling of pathogen response in *Arabidopsis*. *Plant Cell* 17 1196–1204 10.1105/tpc.104.028514 15749761 PMC1087996

